# Increasing HIV counseling and testing services uptake among young people aged 15-24 years, a pilot intervention of soccer activities in Kpaduma II AMAC Local Government Area Federal Capital Territory Abuja Nigeria

**DOI:** 10.1186/2047-2994-4-S1-P112

**Published:** 2015-06-16

**Authors:** FO Agbaje

**Affiliations:** 1 Programs, OROL Youth Empowerment Initiative, Lagos, Nigeria

## Introduction

Uptake of HIV Counseling and Testing (HCT) among young people aged 15-24 years remains relatively low in Nigeria (54.2% for males and 21.3% for females, according to a report published 2004 by Population Council). This low uptake of HCT may hinder access to HIV prevention and care among young people.

## Objectives

The overall objective of this project was to promote uptake of HCT among young people 15-24 years with the use of soccer activities in Kpaduma II AMAC Local Government Area Federal Capital Territory Abuja Nigeria.

## Methods

Data on access and attitudes towards HCT were collected among 273 young people aged 15-24 years living in Kpaduma I and Kpaduma II AMAC Local Government Area Abuja Nigeria. The data were collected using qualitative and quantitative methods. Qualitative data was obtained using key informant interviews and focus group discussions while the quantitative was obtained using structured questions. After the survey, a soccer tournament was organized in the Kpaduma II which is the intervention community with aim to bring youth together to promote the uptake of HCT services. Education sessions on HCT services were organized at the end of each game during the tournament and messages address fear of being positive and stigmatization.

## Results

Out of the 273 respondents who were interviewed in the two communities, (50.4 %) were males and (49.6 %) were females. About (61%) female respondents in Kpaduma I and Kpaduma II communities reported that they had taken an HIV test and (52%) male respondents reported that they had not taken a test. Overall, the female respondents reported highest rate of ever testing and receiving HIV test results. Majority of the respondents who reported that they had not taken a test said they could not take a test due to confidentiality, fear of being tested positive and stigmatization. Over 600 people attended and about 450 tested at the events and received their results and also condoms and information flyers were distributed.

## Conclusion

Soccer as a platform for HIV prevention should be encouraged because it can be harnessed to create sustained behavior change in participants and it’s participatory, team based, and can be used to facilitate both knowledge acquisition.

## Disclosure of interest

None declared.

